# Are health claims in aging-related functional food packages different from those of general functional foods? Content analysis of food packaging from Taiwan

**DOI:** 10.3389/fpubh.2024.1402969

**Published:** 2024-05-30

**Authors:** Chih-Chi Liu, Hung-Chou Lin

**Affiliations:** Department of Adult and Continuing Education, National Taiwan Normal University, Taipei, Taiwan

**Keywords:** aging, consumer protection, functional foods, health claims, older adults

## Abstract

Given the challenges of aging populations, both in Taiwan and globally, issues related to older consumers need more attention. According to surveys in Taiwan, food is one of the most important consumer products for older adults. In recent years, functional foods have become popular, often using health claims as a promotional tool. Therefore, this study has investigated food product packaging in common retail channels in Taiwan by conducting a content analysis of all items with health claims (160 samples). This study specifically compared products related to aging and those unrelated to aging. The results revealed that more than half the participants with health claims did not provide specific descriptions of their health effects. Furthermore, products related to aging often included health terms and claims to supplement with specific nutrients in their health claims. This study has aimed to offer recommendations for educating older consumers, regulating health claims in food advertising, promoting an age-friendly consumer environment, and consumer protection.

## 1 Introduction

As individuals age, their physiological functions gradually decline irreversibly. In light of this reality, public policies should strive to develop effective strategies to enhance the quality of life for the older population. The degeneration of physiological functions also has an impact on cognitive abilities, such as thinking, language, and decision-making. Consequently, communicating product information to older individuals using the same approach as that used by younger consumers may prove insufficient because of their greater cognitive decline of older adults (1). Successful policy formulation for older adults should not solely focus on addressing the needs of disabled older individuals, while ignoring the needs of the majority of older adults (2).

Based on data from Taiwan’s Ministry of the Interior, the older population aged 65 and over accounted for 14.1% of the total population as of the end of March 2018, thereby officially entering the “aged society” phase. It is predicted that by 2025, more than 20% of the population will become a “super-aged society” stage (3). This demographic shift highlights the pressing need to develop comprehensive policies that are related to aging in Taiwanese society. The growing proportion of older adults indicates that societal needs have diverged significantly from those of the past. In response to this trend, the Consumer Protection Committee of the Taiwan Executive Yuan (4) undertook a “Consumption Awareness, Behavior, and Demand Survey Report, which examined the following five primary areas related to senior consumers: consumer behavior and demand, cross-border consumption, consumer disputes, participation in industry sales parties, and consumer awareness and protection. The report aimed to gain insight into the daily consumer behavior and needs of senior consumers and offer pertinent recommendations and policy evaluations based on the report’s findings.

Currently, Taiwan lacks legislation to protect older consumers from the incomplete reception of product information resulting from cognitive decline. This shortcoming can lead to consumer disputes. For example, data from the national consumption behavior survey conducted by the Executive Yuan indicate that only 28.9% of consumers check food packaging labels consistently. However, a survey of older consumer behavior conducted by Executive Yuan (4) revealed that as many as 86% of older individuals tend to scrutinize product labels when purchasing food or packaged drinks. The same survey highlighted that 23.3% of older consumers encountered disputes related to false advertisements or labels, with 26.6% arising from food advertisements. This finding suggests that the product information may influence older consumers when purchasing food, which may result in consumer disputes. Thus, advertising and consumer information strategies should consider the unique characteristics of older adults in order to reduce the possibility of disputes due to weakened cognitive functions, not only among older adults, but also among other ethnic groups.

### 1.1 Potential crisis in regard to food products and health claims advertisements: examining the commodities most needed by older adults in Taiwan

Older adults constitute a significant portion of the consumer market, and food products have emerged as the most commonly purchased goods. A survey in Taiwan revealed that food products accounted for 39.6% of the goods or services that older adults needed or lacked in their lives (4). Furthermore, food items accounted for the highest proportion of goods purchased in person, with 51.3% of older consumers preferring to purchase food products. The popularity of food commodities among older consumers signifies limited business opportunities in the food industry. In the context of in-person purchases, older consumers are exposed to physical product packaging advertisements or advertisements via media channels, such as TV or internet shopping. Health claims in food advertisements are often used as a means of publicity to attract consumers. From the food industry perspective, health claims can enhance product sales and also provide commercial benefits (5, 6). However, it has been suggested that correct product information should be delivered to facilitate future consumption decisions by older consumers. False health claims in advertisements can mislead consumers and affect their decision making.

In 2016, the Fair-Trade Commission of the Executive Yuan released a report highlighting the presence of false advertisements that make people mistakenly believe that a product has a specific function. Similarly, the Food and Drug Administration of the Ministry of Health and Welfare in Taiwan introduced the “Food Labeling Promotion or Advertisement Words Involving Exaggeration, Misunderstanding, or Medical Efficiency Determination Criteria” in the same year to regulate food labeling, publicity, and advertisements that were exaggerated, misleading, or claimed medical efficacy (7). A violation of this regulation would lead to action under the Nutritional Supplement Administration Law. The design of the words and sentences in advertisements may also cause misunderstanding among the consumers (8–10). Therefore, it is important to ensure that advertisements deliver accurate information to consumers. False advertisements and misleading claims in food advertisements can have detrimental effects on older consumers (11, 12), leading to improper decisions and potentially hazardous health outcomes.

### 1.2 Application context of the health claims: the advertisement of functional food

The concept of functional foods has become increasingly popular in recent years (13–15), as people have become more health-conscious and seek ways to improve their well-being. Doyon and Labrecque (16) proposed the following definition of functional foods through literature review and Delphi method with experts:

“*A functional food is, or appears similar to, a conventional food. It is part of a standard diet and is consumed on a regular basis, in normal quantities. It has proven health benefits that reduce the risk of specific chronic diseases or beneficially affect target functions beyond its basic nutritional functions.*”

Additionally, functional foods are also considered to provide additional health benefits beyond basic nutrition (13). Health claims are often used in the marketing of these types of foods (17–19), as they highlight the potential health benefits and can influence consumer behavior (20).

Health claims are any statements or claims suggesting that a food has specific health benefits or can prevent or treat a disease (21, 22). Health claims are used in packaging, advertising, and other promotional materials. Various national and international bodies regulate the use of health claims, including the European Food Safety Authority (EFSA) and the U.S. Food and Drug Administration (FDA). In the European Union, health claims are subjected to a rigorous scientific evaluation process before they can be used in marketing (23).

Functional foods are often marketed using health claims, because these claims can help distinguish them from other types of food and therefore attract health-conscious consumers (24, 25). For example, products high in antioxidants may be marketed as having anti-aging properties (26), whereas those high in omega-3 fatty acids may be marketed as good for heart health (27). Health claims can also be used to differentiate between the different types of food within a category, such as between different types of yogurt or cereal. Another health claim strategy used in functional food advertisements is the use of scientific-sounding language. Food companies may use terms such as “clinically proven” or “scientifically tested” to give the impression that their products have been rigorously tested and proven effective (28). However, consumers may not always understand these terms and may be misled into thinking that a product is more effective than it is (29). Celebrity endorsements are common health claim strategies used in functional food advertisements. Companies may use a celebrity to promote their products and claim that the celebrity uses these products to maintain health. This can be an effective strategy to create a positive association with a product and increase consumer interest in it (30). However, consumers may also be misled into thinking that the product is more effective than it actually is or that celebrity endorsement guarantees its effectiveness (31, 32). Finally, food packaging may include health claims regarding nutritional components. For example, a product may be advertised as “low-fat” or “sugar-free” (33). While these health claims are accurate, they may not provide the necessary information for all types of consumers’ dietary choices (34). Since different consumers prioritize different health claims, a more nuanced exploration of the content of health claims on food packaging is needed to meet the diverse needs of consumers.

The use of health claims in marketing can significantly impact consumer behavior. Previous research has shown that health claims can influence the consumers’ perceptions of a product’s health and nutritional value, and can affect their willingness to pay for it. However, the use of health claims can be misleading or confusing for consumers, particularly if the claims are not supported by scientific evidence or are vague or ambiguous. In order to address these issues, regulatory bodies have established guidelines on the use of health claims in food marketing. These guidelines typically require health claims to be supported by scientific evidence, clear and specific, and not misleading or confusing to consumers (23). The guidelines also require health claims to be used in a way that is unlikely to encourage the overconsumption of food or create a false impression of its healthiness.

Health claims play a significant role in the marketing of functional foods. Although health claims can help distinguish between these types of foods and attract health-conscious consumers, they can also be misleading or confusing if not used properly. Regulatory bodies have established guidelines for the use of health claims in food marketing to ensure that they are supported by scientific evidence, are clear and specific, and are unlikely to encourage overconsumption or create a false impression of a product’s health. Therefore, based on the products that older adults in Taiwan most commonly purchase personally, which are likely to be food, and considering the potential for misunderstanding health claims in food advertising by older adults, there is a risk of health and consumption disputes in this demographic. Thus, this study aimed to conduct a content analysis of the packaging of food products with health claims that are commonly sold in consumption channels frequented by older adults. This study also explored whether health claims related to aging differ from general health claims. The anticipated results of this study aim to provide future researchers and practitioners with an analytical framework to further explore the consumption behavior of older adults regarding such products and consumer protection.

## 2 Materials and methods

### 2.1 Sampling

Based on government surveys conducted in the researcher’s location (Taiwan), it has been indicated that older adults most commonly purchase food products in person. Therefore, this study has sampled paper catalogs of products from local supermarkets, including the top two retailers in terms of market share (the market share of the third-ranked supermarket was below 1%) (35). Convenience stores were not sampled mainly because of their limited product variety, and most of the products they sold were also available in supermarkets. Additionally, the study excluded hypermarkets because, although they may have the most extensive variety of products sold directly to consumers, older adults might find it challenging to purchase a large quantity of products at once due to potential physical limitations. The sampling period for this study was from June 1, 2023, to July 6, 2023, with the catalog validity period for the top-ranked supermarket from June 16, 2023, to July 6, 2023, and for the second-ranked supermarket from June 1, 2023, to June 27, 2023. The study did not sample products from earlier periods as they might include items that have been discontinued. Furthermore, the supermarket ranked first in market share, as mentioned in Shieh et al. (36), is distributed across 86.7% of administrative districts in Taiwan, including some sparsely populated island and rural areas. This, to a certain extent, ensures the representativeness of the sampling in the current study. After excluding duplicate products from both channels, the top-ranked supermarket’s paper catalog contained 459 food products and the second-ranked supermarket contained 201. Finally, the study’s methodology and design aimed to understand whether there were differences in packaging strategies for food products with and without health claims related to aging. In this context, the two researchers initially classified all of the sampled food products into those with and without health claims. This initial categorization was determined through a joint discussion between the two researchers. Finally, we obtained 138 food products with health claims from the paper catalogs of the top-ranked supermarket and 22 from the second-ranked supermarket, for a total of 160.

### 2.2 Research design and methods

This study primarily employed content analysis of food product packaging in supermarket paper catalogs. The aim was to systematically classify and describe data based on predefined coding categories to obtain structured and quantitative information about the content. Content analysis was used to quantitatively transform the qualitative data through coding for statistical analysis. The design steps for the coding categories included defining the research question, a literature review, coding category design, coder training and initial testing, calculating the inter-rater reliability, executing the coding, and data analysis (37). The design of the coding categories was based on the results of the research question and literature review, creating a set of coding categories that were clear, specific, and operational to classify the data into corresponding categories. Based on the literature review, six coding themes for digital game cognition were synthesized: (1) specific diseases, symptoms, or effects; (2) endorsers; (3) third-party endorsements; (4) scientific evidence; (5) health terminology; and (6) specific nutrients or ingredients. The definitions, examples, and literature backgrounds of the detailed categories are listed in Table 1. Subsequently, based on Table 1, the two researchers independently coded 160 items.

**Table 1:** The coding themes, item and theoretical background.

Themes	Item	Theoretical background
Specific diseases, symptoms, or effects	Related to aging	Migliore et al. (27) and Wilson et al. (26)
	Not related to aging	
	Not specifying any diseases, symptoms, or effects	
Endorsers	Celebrity	Freiden (40), Kusumasondjaja & Tjiptono (31), and Muela-Molina et al. (32)
	Amateur	
	Expert	
	Without any endorsers	
Third-party endorsement	Containing award	Dean and Biswas (41)
	Containing organizational certification	
	Without any third-party endorsements	
Provide scientific evidence	Narrative evidence	Hill et al. (42) and Shahidi (28)
	Statistical evidence	
	Contains both narrative and statistical evidence	
	No provided	
Health terminology	Contains health terminology	Berry et al. (29)
	Without health terminology	
Specific nutrients or ingredients	Containing specific ingredients or nutrients	Barauskaite et al. (13)
	Without specific ingredients or nutrients	

### 2.3 Data analysis

The purpose of this study’s content analysis was to systematically classify and describe data based on predefined coding categories to obtain structured and quantitative information about the content. Content analysis was conducted on 160 food packaging items. First, a coding table was established based on the literature review, as shown in Table 1. Each product packaging item is coded once for each of the six themes.

Two academically trained researchers served as coders in the study. After the coding was completed, an inter-rater reliability analysis was conducted. Following Neuendorf (38) recommendation, and by considering the potential influence of coder subjectivity on the scoring of research variables, this study randomly selected a sample of over 10% of the data. From this sample, 20 items were selected to calculate the inter-rater reliability of the two coders. Kappa consistency coefficient analysis was used for reliability analysis. The results showed Cohen’s kappa = 0.89, with internal consistency coefficients for coding exceeding 0.8, indicating reliability (39). Finally, the two coders reached a consensus through discussion to analyze the results of the content analysis.

## 3 Results

This study conducted a content analysis of the coding themes in Table 1. In the first step, all food packaging was categorized into three groups according to the coding theme “Specific Diseases, Symptoms, or Effects”: related to aging, not related to aging, and not specifying any diseases, symptoms, or effects. Among the 160 food product packages, there were 29 items related to aging, 50 were not related to aging, and 81 did not specify any diseases, symptoms, or effects. This indicates that in Taiwan’s retail channels, most of the food products with health claims do not specify any diseases, symptoms, or effects.

**Figure 1:**
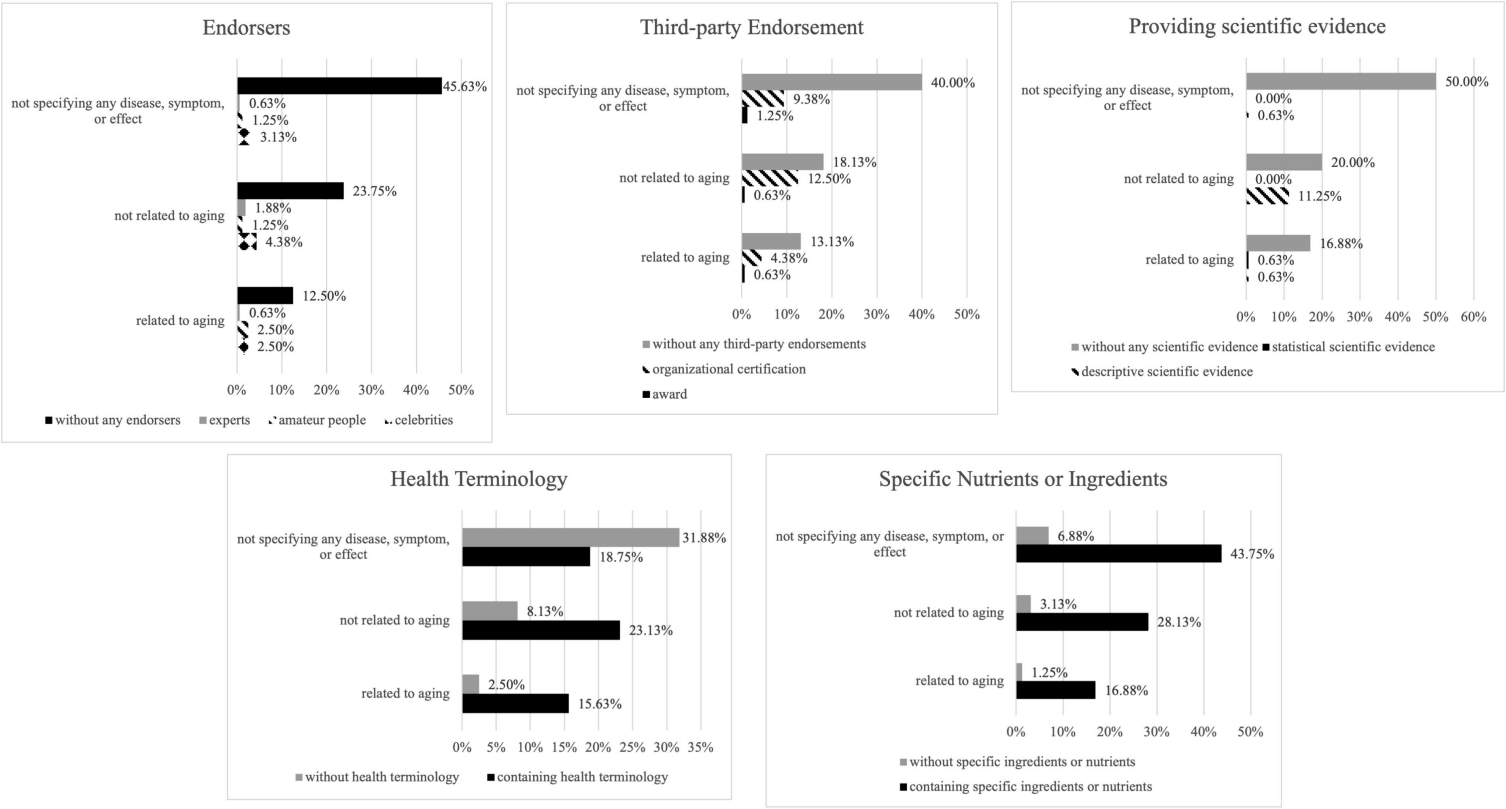
The visualized coding results.

Subsequently, all other coding themes were cross-compared with the aforementioned themes. However, because of disparate distribution proportions, with many categories having counts below five or even zero, conducting a chi-square analysis was not appropriate (43). Therefore, the data analysis in this study presents the distribution results and percentages. The study then proceeded to further analyze the distribution under each coding theme. It is noteworthy that most of the functional foods do not specify any disease, symptom, or effect on their packaging. Additionally, when health claims specifically target diseases, symptoms, or effects unrelated to aging, they mostly provide scientific evidence. Finally, when health claims are related to aging, the most commonly used strategy appears to be labeling health terms or specific nutrients. The results are presented in Table 2 and Figure 1.

**Table 2:** The coding results.

	Celebrities	Amateur	Experts	Without any endorsers	Total
Related to aging	4 (2.50%)	4 (2.50%)	1 (0.63%)	20 (12.50%)	29 (18.13%)
Not related to aging	7 (4.38%)	2 (1.25%)	3 (1.88%)	38 (23.75%)	50 (31.25%)
Not specifying any disease, symptom, or effect	5 (3.13%)	2 (1.25%)	1 (0.63%)	73 (45.63%)	81 (50.63%)
Total	16 (10.00%)	8 (5.00%)	5 (3.13%)	131 (81.88%)	160 (100.00%)

	Award	Organizational certification	Without any third-party endorsements	Total
Related to aging	1 (0.63%)	7 (4.38%)	21 (13.13%)	29 (18.13%)
Not related to aging	1 (0.63%)	20 (12.50%)	29 (18.13%)	50 (31.25%)
Not specifying any disease, symptom, or effect	2 (1.25%)	15 (9.38%)	64 (40.00%)	81 (50.63%)
Total	4 (2.50%)	42 (26.25%)	114 (71.25%)	160 (100.00%)

	Descriptive scientific evidence	Statistical scientific evidence	Without any scientific evidence	Total
Related to aging	1 (0.63%)	1 (0.63%)	27 (16.88%)	29 (18.13%)
Not related to aging	18 (11.25%)	0 (0.00%)	32 (20.00%)	50 (31.25%)
Not specifying any disease, symptom, or effect	1 (0.63%)	0 (0.00%)	80 (50.00%)	81 (50.63%)
Total	20 (12.50%)	1 (0.63%)	139 (86.88%)	160 (100.00%)

	Containing health terminology	Without health terminology	Total
Related to aging	25 (15.63%)	4 (2.50%)	29 (18.13%)
Not related to aging	37 (23.13%)	13 (8.13%)	50 (31.25%)
Not specifying any disease, symptom, or effect	30 (18.75%)	51 (31.88%)	81 (50.63%)
Total	92 (57.50%)	68 (42.50%)	160 (100.00%)

	Containing specific ingredients or nutrients	Without specific ingredients or nutrients	Total
Related to aging	27 (16.88%)	2 (1.25%)	29 (18.13%)
Not related to aging	45 (28.13%)	5 (3.13%)	50 (31.25%)
Not specifying any disease, symptom, or effect	70 (43.75%)	11 (6.88%)	81 (50.63%)
Total	142 (88.75%)	18 (11.25%)	160 (100.00%)

## 4 Discussion

### 4.1 Most of the functional foods do not specify any disease, symptom, or effect on their packaging

Based on the current research coding results, over half of the functional food packaging in the samples did not claim specific effects or impacts on any disease or symptom. These functional food packagings that do not specify any effects mostly use general phrases such as “become healthier,” “make you healthier,” or “simple ingredients,” “natural ingredients” to persuade consumers that their products may be beneficial to health after purchase or use. However, in reality, it does not specify the effects or changes that can be achieved. A systematic review of food packaging also noted the continuous use of health-related arguments across all types of food packaging as sales claims, regardless of whether these foods are healthy, beneficial, useful, or truthful (44). Previous studies have also indicated that the food advertisements claiming simple ingredients are more appealing to older consumers, and some findings align with the current research (45). Accordingly, contemporary functional food products may be more inclined to undergo less artificial processing or use fewer chemical additives in order to demonstrate their health benefits rather than specifying concrete effects on packaging.

This phenomenon raises the question of whether certain food packaging products may provide vague health claims to avoid legal risks, although these products may have no actual impact on health, leading consumers to believe that these products may be more effective than they actually are. Although the current guidelines (23) require health claims to be clear, specific, and non-misleading to consumers, if local governments or enforcement agencies do not enforce these standards, consumers may still be at risk. Therefore, the relevant agencies worldwide should actively consider this issue and establish policies and regulations that provide the best public protection.

### 4.2 Noticeable differences in the coding distribution trends: providing scientific evidence

Some food companies may claim in their product packaging that their products have undergone scientific testing or have provided relevant clinical trial evidence (28). From the current research results, it can be observed that in samples where health claims specifically mention diseases, symptoms, or effects, products unrelated to aging include more descriptive scientific evidence than products with health claims related to aging. The packaging of these functional foods often includes claims such as “scientifically proven,” “animal experiments show significant improvement,” and some even attach references to support their statements. These claims are mostly presented as descriptive statements and rarely provide statistical data. This may be related to past research indicating that consumers do not always comprehend the meaning of such data (29), leading to the current research findings.

However, this phenomenon seems to be less prevalent in the health claims related to aging foods, possibly because the cognitive functions of older adults may decline compared to younger consumers, making the same claims approach as young consumers unsuitable for older adults (1). However, this does not necessarily imply that older adults value this information. Future health claims should consider how to provide content that is more easily understandable to older adults to meet the information acquisition needs of the relevant consumer group. This may not only reduce the likelihood of consumer disputes but also promote the protection of older consumers.

### 4.3 Common strategies for the health claims related to aging on food packaging: health terminology and specific nutrients or ingredients

Finally, the current research findings reveal that, among samples containing health claims related to aging, the most commonly used strategies seem to involve the use of health terminology and emphasis on specific nutrients. Similar to previous studies that have indicated that antioxidant-related components being claimed to prevent aging (26), the current research also identifies a similar phenomenon. In the present study, the most frequently used nutrient claimed by the aging-related products was “calcium.” This may be related to numerous studies on aging suggesting calcium loss or symptoms related to osteoporosis in older individuals (46). In summary, this may indicate that older adults favor health claims that emphasize specific nutrients or terms, potentially leading food manufacturers targeting this demographic to preferentially use such strategies to promote their products. Therefore, it is suggested to promote the role of relevant nutrients in the health of older adults from the perspective of health education or policy advocacy, thus enabling older consumers to make better choices regarding related products.

## 5 Conclusion

This study investigated health claims regarding functional food packaging in the Taiwanese market and summarized the strategies commonly used to promote these claims. This study specifically categorized the health claims of these functional foods as related or unrelated to aging. Given the global challenge of an aging society, the current research aimed to explore products (foods) that older adults are likely to consume frequently, as the food is closely associated with health. Furthermore, this aligns with previous research indicating the necessity for conveying product types and their corresponding features more accurately (47). It is hoped that future researchers and practitioners will develop more comprehensive and effective health claims regulations and strategies based on the results of this study, establishing a society and consumer environment that is friendly to individuals of all ages.

## Author’s note

Some content of this article is derived from the unpublished doctoral dissertation of the first author, C-CL.

## Data availability statement

The raw data supporting the conclusions of this article will be made available by the authors, without undue reservation.

## Author contributions

C-CL: Conceptualization, Data curation, Formal analysis, Funding acquisition, Investigation, Methodology, Resources, Software, Validation, Visualization, Writing – original draft, Writing – review & editing. H-CL: Project administration, Supervision, Writing – review & editing.
